# Role of Microenvironment in Glioma Invasion: What We Learned from In Vitro Models

**DOI:** 10.3390/ijms19010147

**Published:** 2018-01-04

**Authors:** Ivana Manini, Federica Caponnetto, Anna Bartolini, Tamara Ius, Laura Mariuzzi, Carla Di Loreto, Antonio Paolo Beltrami, Daniela Cesselli

**Affiliations:** 1 Department of Medicine, University of Udine, P.le Kolbe 4, 33100 Udine (UD), Italy; ivana.manini@tiscalinet.it (I.M.); the_capo@hotmail.com (F.C.); anna.bartolini90@hotmail.it (A.B.); laura.mariuzzi@uniud.it (L.M.); carla.diloreto@uniud.it (C.D.L.); antonio.beltrami@uniud.it (A.P.B.); 2 Neurosurgery Department, University Hospital of Udine, P.le S. Maria della Misericordia, 33100 Udine (UD), Italy; tamara.ius@gmail.com; 3 Pathology Department, University Hospital of Udine, P.le S. Maria della Misericordia, 33100 Udine (UD), Italy

**Keywords:** glioblastoma, invasion assay, personalized medicine, glioma-associated stem cells, microfluidic systems, 3D culture, mixed culture, tumour microenvironment

## Abstract

The invasion properties of glioblastoma hamper a radical surgery and are responsible for its recurrence. Understanding the invasion mechanisms is thus critical to devise new therapeutic strategies. Therefore, the creation of in vitro models that enable these mechanisms to be studied represents a crucial step. Since in vitro models represent an over-simplification of the in vivo system, in these years it has been attempted to increase the level of complexity of in vitro assays to create models that could better mimic the behaviour of the cells in vivo. These levels of complexity involved: 1. The dimension of the system, moving from two-dimensional to three-dimensional models; 2. The use of microfluidic systems; 3. The use of mixed cultures of tumour cells and cells of the tumour micro-environment in order to mimic the complex cross-talk between tumour cells and their micro-environment; 4. And the source of cells used in an attempt to move from commercial lines to patient-based models. In this review, we will summarize the evidence obtained exploring these different levels of complexity and highlighting advantages and limitations of each system used.

## 1. Introduction

Gliomas represent the main type of malignant brain tumours diagnosed in adults, being glioblastoma (GBM) the most malignant and incurable form [1]. Despite the aggressiveness of the treatment, involving radio- and chemo-therapy after a maximal surgical resection, GBM patients show a median survival of 14 months [2]. One of the peculiarity of GBM, hindering the efficacy of treatments, is the diffuse invasion of tumour cells into the surrounding brain [3]. Although the infiltration usually produces recurrences within 1–2 cm from the original tumour mass, appearing few months after the first diagnosis (methachronous lesions) or being already present at the time of the initial presentation (synchronous gliomas) [3,4], single infiltrating cells are often spread throughout the entire brain parenchyma [5]. Moreover, although characterized by a better prognosis, also 70% of WHO grade II gliomas undergo recurrence and anaplastic transformation within 10 years from the first surgery [6,7], making the invasive properties of glioma cells a more general problem, somehow independent from the tumour grade [8].

For this reason, understanding the mechanisms of glioma invasion could open the way to new treatments able to revert the severe prognosis of GBM and, possibly, to cure low-grade gliomas [9].

This objective requires both knowing how gliomas invade the brain and deciphering the molecular mechanisms that underlie it. The first objective has been relatively achieved thanks to numerous neuropathological studies, conducted both in animal and human tissues. These investigations have benefited, over the years, from innovative techniques for the in-situ identification of cancer cells and for the visualization of both their trafficking and relationships with adjacent structures. The second aim has only been partially attained because it requires the development of experimental models that allow defining molecular mechanisms through the ability to interfere with the system. Although in vivo animal models allow studying GBM within the microenvironment of a living host, they are complex, expensive, time consuming, often impaired by a low reproducibility and make it difficult to dissect the different components [10]. Additionally, for the principle of the 3R (reduce, refine and replace), animal experiments are allowed when they cannot be replaced by alternative methods. From the other site, in vitro assays allow to explore specific aspects but they cannot take into account all the complexity of the in vivo system [10]. However, in these years, progresses in cell biology, the development of three-dimensional (3D) cell cultures and the introduction of new micro- and nano-technologies have led to the development of innovative in vitro models aimed at increasing their ability to integrate the numerous variables affecting the in vivo invasion of glioma cells [11].

In this review, after a brief description of the modalities of glioma invasion and of the actors involved, we will critically present the in vitro assays that have been developed so far, summarizing results and exploring advances and limitations.

## 2. Glioma Cell Invasion: The Picture of a Complex Event

This section will describe the essential knowledge about glioma invasion to highlight what are the key features that in vitro assays should incorporate to mimic the in vivo invasion.

### 2.1. How and Where Glioma Cells Move

Unlike other malignant solid cancers, gliomas rarely metastasize outside the brain and glioma cells do not use intravascular or lymphatic routes to migrate [12]; instead, they move through the extracellular space of the brain tissue [8,9,13,14]. This invasion process differs from that of migration because cell movements require glioma cells to cross tissue barriers by both adhering and degrading the extracellular matrix (ECM) and remodelling their cytoskeleton and cell volumes [8,9,14].

It is well known that cancer cells can invade either as individual cells, using mesenchymal or amoeboid movement, or disseminate from the original tumour mass organized in clusters or sheets (collective migration or “Roman army” movement) [8,15,16]. Glioma cells mainly use the first type of migration, also defined as a “guerrilla war,” one of the most efficient method that cancer cells adopt to migrate away from the main tumour bulk [17]. This single tumour cells, undetectable by the most sophisticated diagnostic imaging techniques, can be therefore responsible for the formation of tumour recurrences. This type of invasion is reminiscent of that of neural stem cells during the embryonic development or during the repairing processes in damaged adult brain tissues [18].

**Figure 1:**
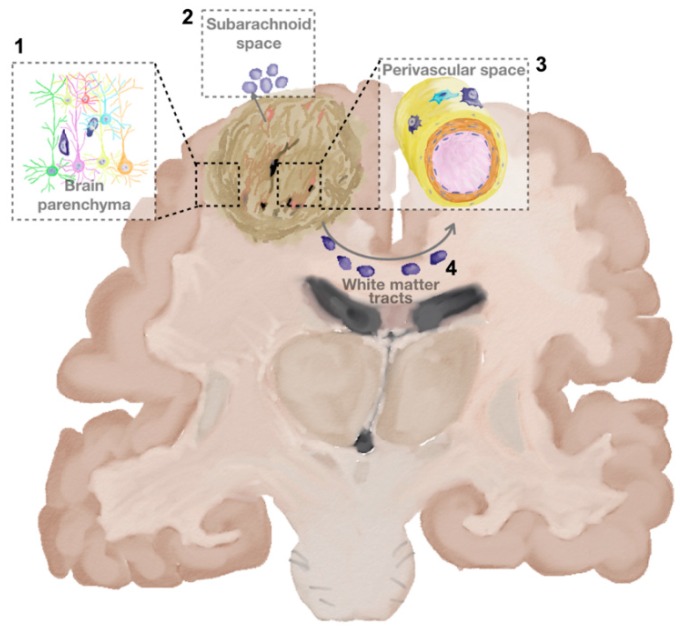
Preferential migration routes of glioblastoma cells. Tumour cells (dark blue) tend to invade the brain along pre-existing brain structures, such as interstitial spaces of the brain parenchyma (1); the subarachnoid space (2); the perivascular space (3) and white matter traits (4).

The glioma cell invasion shares, with that of neural stem cells, other two key features. First, they tend to migrate along pre-existing brain structures, such as interstitial spaces of the brain parenchyma, blood vessels, white matter traits and the subarachnoid space (Figure 1) [9]. This observation was first described, in 1938, by a German neuropathologist, Hans Joachim Scherer and, for that reason, these migratory pathways are frequently called “Scherer’s structures” [19]. These latter oppose distinctive physical and mechanical barriers to glioma cell invasions. For example, in the brain parenchyma, neuronal and glial cell processes and bodies design narrow extracellular spaces providing a great resistance to glioma cell movements; while, in the perivascular space, tumour cells tend to displace astrocyte endfeet [20]. The pathways involved in these different migration routes, although not fully elucidated yet, seem to be distinctive and, possibly, related to the second features shared by glioma cells and neural stem cells, that is their sensitivity to neurotransmitters and neuropeptides, as well as to extracellular matrix components and adjacent cells [9]. The knowledge of these different mechanisms is fundamental in the perspective of designing drugs able to target invasion, since more than one intervention can be required to stop these different invasion roads.

### 2.2. The Actors Involved in Glioma Cell Invasion

**Figure 2:**
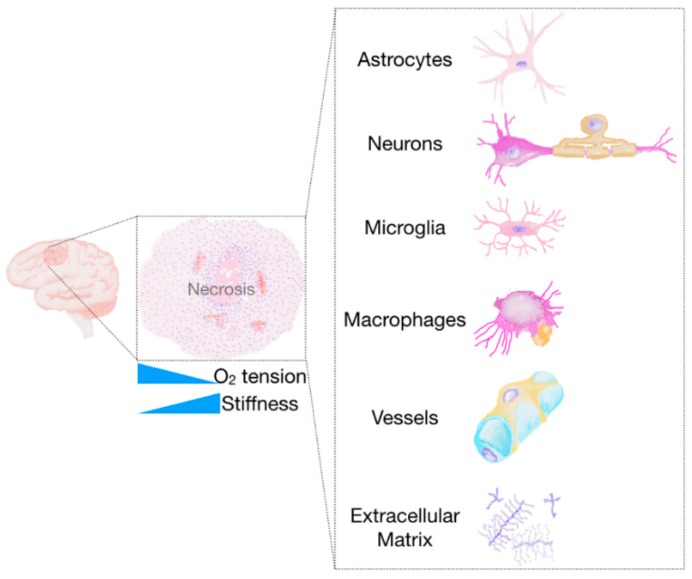
The glioma microenvironment. The glioma microenvironment consists, besides of tumour cells, of different cell types (including endothelial cells, astrocytes and microglia) as well as the extracellular matrix (ECM). The presence of necrotic areas can cause oxygen (O_2_), pH and nutrient gradients. Additionally, tumour regions are characterized by an increased stiffness with respect to the surrounding brain parenchyma. Rare stem cells (NSC, GASC and MSC) and interstitial flow, although not depicted here, paly also a role in the glioma microenvironment.

Accumulating evidences are depicting a multifaceted scenario in which not only an increasing number of actors seems to take part to the invasion process (e.g., tumour cells, extracellular matrix components, non-tumour brain cells) (Figure 2) but these actors change depending on the invasion path. However, the mechanisms involved in the various invasion routes could find a common denominator in the so-called hydrodynamic model of cell invasion [9].

#### 2.2.1. Tumour Cells

Independently from the invasion route, glioma cells show a mesenchymal type of migration and must often cross extracellular spaces smaller than their nucleus. Specifically, glioma cells become fibroblast-like and polarized, with leading and trailing edges located at the opposite sites of the cell body. The high polymerization of actin filaments extends the cell membrane outward, at the leading edge (pseudopod), which interact with the ECM through clustered integrins present at the cell membrane. Integrins recruit adaptor molecules and signalling proteins, via their cytoplasmic domains, triggering signals into the cell (phosphorylation/de-phosphorylation via focal adhesion kinase, FAK) [21]. Afterwards, surface proteases (i.e., membrane-type matrix metalloproteinases, MT-MMPs) are recruited at the focal contacts with the ECM and start to produce soluble matrix metalloproteases (e.g., MMP-2 and MMP-9) able to degrade and remodel the surrounding ECM, favouring the invasion [22]. Finally, cell contracts itself, by the acto-myosin complex engagement, leading to focal contact disassembly, integrin recycling and detachment of the trailing edge [23].

Regarding the nature of invading cells, glioma stem cells (GSC) have been reported as the putative population responsible for glioma invasion [24]. Since endowed with self-renewal properties, they can initiate and maintain tumour growth [25,26] and can be responsible for tumour recurrence [27,28]. GSC are profoundly influenced by the microenvironment in which they reside [29]. In fact, the bidirectional cross talk between GSC and the tumour microenvironment (TME) regulates not only their stem cell properties but promotes proliferation, angiogenesis and invasion [30].

#### 2.2.2. The Tumour Microenvironment

Invasion is considered one of the hallmarks of cancer [27,29]. As widely accepted, tumour hallmarks could not be perceived as a tumour-sustaining entity but instead they are the result of the continuous bi-directional interaction between tumour cells and their surrounding microenvironment [27,29,31]. Tumour cells activate the microenvironment and the activated microenvironment acts supporting the tumour growth and favouring, among others, tumour invasion [27,29]. The glioma microenvironment is a dynamic entity that consists, besides of glioma cells (including GSC), of an intricate network that encompasses different cell types (e.g., endothelial cells, astrocytes and microglia), stromal components, soluble factors as well as the ECM [32,33]. Altogether, these entities participate not only in gliomagenesis but in tumour growth and progression [33].

##### The Extracellular Matrix

As mentioned, when glioma cells move through the brain parenchyma, they have to establish contact with molecules of the ECM and components of the basement membrane. The brain parenchyma shows a unique composition, lacking the fibrillar and rigid matrix composed of collagen, fibronectin and laminin, typical of other tissues [20]. Instead, brain intercellular spaces are filled by proteoglycans [20,34,35], a class of water binding proteins produced by astrocytes and oligodendrocytes, hyaluronan (HA) [36,37,38,39] and tenascin C [34]. These proteins confer to the brain a gelatinous consistency, not found elsewhere [20]. Indeed, many of these proteins are specific for the brain and can act favouring cell invasion (brevican, neurocan and phosphocan) [35]. Additionally, brain tumours are often associated with an enhanced production of pro-invasive HA [36,37,38,39] and tenascins [35,40,41]. GSC invasiveness has been related to the binding of either CD44 or receptor for hyaluronate-mediated motility (RHAMM) to HA [36,37,38,42]. As already mentioned, GSC can remodel the ECM favouring invasion through the release of MMP, such as MMP2 and MMP9, released upon toll like receptor 2 (TLR2) activation [43] and MMP13 [44]. The ADAM (a disintegrin and metalloproteinase) proteins, especially ADAM17 and ADAM9 [45,46,47] and cysteine cathepsins are also involved in glioma migration [48].

Importantly, glioma cells are not only influenced by the biochemical composition of the ECM but their motility is powerfully affected by biophysical inputs, i.e., density, rigidity and geometry of the ECM [49,50]. Ulrich and co-workers investigated the role of the ECM rigidity in stimulating the migration of glioma cells, in vitro. They showed that, upon increasing ECM stiffness, glioma cells are able to form stress fibres and focal adhesions more rapidly than in lower rigid substrates and they migrate quickly. This process seems to depend on the non-muscle myosin II [51].

Blood vessels represent another preferential substrate for glioma cell migration. Specifically, tumour cells do not intravasate inside the vessels but they associate with the vascular walls and migrate along them. It was shown, in vitro and in vivo, that brain vascular endothelial cells secrete bradykinin, a chemotactic signal attracting glioma cells [13]. Bradykinin binds to its receptor (BR-2) on the glioma cell surface and activates GPCR signalling and the subsequent IP3-R-dependent release of Ca^2+^ from intracellular stores [52]. Changes in Ca^2+^: affect the cell movement because influencing the actin-myosin mediated contraction [53]; regulate the cytoskeleton influencing the dynamics of tubulin; and control the focal adhesion kinases, thus altering the adhesion of cells to substrates [54].

The movement of glioma cells along the surface of blood vessel alters the physiological organization of the brain vasculature, in which astrocytes endfeet are in close association with the endothelial cells in the vascular walls [55]. Astrocytes endfeet and endothelial cells are both anchored to one or two layers of basement membrane. The astrocytes-vascular interface is important for the brain homeostasis, to regulate both blood flow and neuronal activity and to maintain the blood-brain barrier (BBB) [56]. When glioma cells migrate, they displace the astrocytes endfeet by degrading the basement membrane surrounding blood vessels [55,57]. This perturbation leads to the breakdown of the BBB, disruption the neurovascular unit and abolishment of the regulation of blood vessel diameter by astrocytes [55]. On the other hand, glioma cells can have an easy access to oxygen and nutrients from the bloodstream [55].

##### Interstitial Flow

Other important players driving glioma invasion are chemokine gradients and the interstitial flow [58]. This latter can be defined as the result of the difference between the interstitial pressure of the tumour, which is higher and the normal interstitial pressure of the healthy brain tissue. The difference in pressure makes the tumour exude the fluid in the brain parenchyma, increasing fluid flow up to 100 times [59]. This phenomenon directly correlates to glioma growth [11]. In fact, conversely to normal brain fluid flow and drainage, in gliomas cerebral fluid tends to accumulate, causing a higher interstitial pressure that triggers a cascade of events, such as mechanotrasductional changes (ECM stiffening, for instance) and expansion of the extracellular space and increasing cell growth [11]. Specifically, the pressure-driven invasion appears to be mediated by the CXCL12-driven chemotaxis (through CXCR4) [58]. Munson et al., in fact, reported that the altered flow, generated after the tumour growth, is able to create a gradient of CXCL12, from the tumour bulk to the tumour border. A fraction of cells located at the border express the receptor CXCR-4 and secrete CXCL12, so they are stimulated to migrate by a phenomenon referred to as autologous chemotaxis [58].

##### Hypoxia and Acidosis

One of the most important features of GMB, strictly related to the presence of necrotic areas [60,61,62], is its heterogeneous hypoxic and acidic environment that is fundamental for both the maintenance of GSC self-renewal and the induction of glioma invasion [24,30]. Hypoxia and acidosis are also able to trigger a stem cell program in non-stem cell tumour cells [63,64,65,66]. Specifically, the differential hypoxic environment in the different regions of the tumour is able to stimulate the expression of the transcription factors hypoxia-inducible factor (HIF) HIF1α and HIF2α, able to activate a stem cell program [67] and favour an invasive phenotype [64,65], partly mediated by Notch signalling [68,69].

Similarly, acidosis, linked to hypoxia and increased glioma metabolism [70], is able to trigger a stem cell program [66] and acts through the heat shock protein 90 to promote HIF function [71]. Moreover, acidosis induces in glioma cells a compensatory up-regulation of the sodium–hydrogen exchanger isoform 1, protein that favour glioma invasion by influencing MMP activity and directly interacting with ezrin [72].

##### Non-Neoplastic Cells of the Glioma Microenvironment

Many cell types are known to infiltrate the GBM mass such as microglia, macrophages, astrocytes, endothelial cells and stem cells [29].

*Microglial cells* constitute about 40% of the tumour mass [24]. They play a key role in glioma invasion through several mechanisms [73,74]. GSC are able to recruit and activate microglial cells [75] and, through the release of IL6, favour their pro-invasive action [76]. Glioma cells are also able to activate toll-like receptor (TLR) signalling in the microglia, which results in MT1-MMP expression and subsequent activation of the pro-invasive MMP2 by GSC [77,78]. Microglia cells are also able to favour glioma invasion by releasing of many growth factors and ECM proteins [79] and thus activating, in glioma cells, several pro-invasive signalling pathways, including: protein tyrosine kinase 2 beta (Pyk2) signalling [80,81,82], osteopontin-CD44 signalling [83], epidermal growth factor (EGF) signalling [84] and transforming growth factor-β (TGF-β) pathways [85,86,87,88,89]. *Tumour associated macrophages* (TAM), are circulating monocytes recruited into the tumour microenvironment where they are skewed to an M2 phenotype [90,91,92,93]. Besides acting by favouring immune-escape, they are also able to modify glioma cells by releasing IL6 and IL10 [90,91,92,93]. Other immune cellular subtypes involved in gliomas are monocytes, neutrophils and myeloid-derived suppressor cells (MDSC), which are frequently present in the tumour microenvironment. These cells are known to take part to angiogenesis, immune-escape, drug resistance and invasion [30].

*Astrocytes* are also considered important participants not only in the gliomagenesis but also in the tumour progression and invasion [33,94]. They act on GSC either by direct cell contact or by releasing proteins associated with cell invasion, such as chemokines and cytokines, including IL6 and TGFβ2, or MMP2 [24,95,96,97].

One of the most important participants in the perivascular niche are the neighbouring *endothelial cells* (EC). These cells are recruited with the release of high levels of proangiogenic factors, such as vascular endothelial growth factor (VEGF), from the tumour, which exploits EC in order to promote tumour growth and angiogenesis [29]. Conversely, EC release soluble factors such as transforming growth factor-β (TGF-β) and platelet derived growth factor (PDGF) for GSC survival, nitric oxide- (NO-) cyclic GMP and Notch for maintenance of GSC stemness and self-renewal capacity [30]. Interestingly, Liu et al. showed that the activation of the angiopoietin 1 (Ang1)/Tie2 cross talk between glioma cells and endothelial cells was paralleled by an increase in glioma invasion [98].

The TME is also characterized by the presence of non-tumour stem cells, including neural stem cells, mesenchymal stem cell [99,100] and glioma-associated stem cells [101].

*Mesenchymal stem cells* have been isolated from both murine [99] and human gliomas [100]. In a murine model, infiltration of brain tumour MSC correlated to tumour progression [99]. Similarly, glioma associated human MSC increased proliferation and self-renewal of GSC in vitro and increased their in vivo tumorigenicity by secreting interleukin-6, which activates STAT3 in GSC [100].

*Glioma associated stem cells* (GASC) represent a population of stem cells isolated from human gliomas [101]. These cells presented a mesenchymal surface immunophenotype, aberrant growth properties and were able to support, in vitro, both GSC migration and proliferation through the release of exosomes [101]. The phenotype of GASC could predict patient prognosis, thus supporting the notion that they could represent a patient-based in vitro model of the glioma microenvironment [101].

## 3. Evolution of In Vitro Models to Study Glioma Invasion

Until now, the pivotal problem to further explore glioma invasion mechanisms and develop new therapies is to replicate in vitro the complex structural organization of the brain. Specifically, different elements have been shown to play a role: (1) the mechanism of invasion; (2) the resident brain structures used by the tumour cells to migrate (e.g., intraparenchymal or perivascular routes); (3) the type of tumour cells; (4) the different elements of the TME (e.g., tumour supporting cells, chemotactic gradients, ECM composition and stiffness) influencing the migration; (5) the interstitial flow.

For this reason, models have been developed with the goal of: 1. switching from two-dimensional (2D) models to three-dimensional (3D) models (Table 1); 2. using multiple cell types and exploring the effects of cross-talk between cancer cells, TME cells and ECM elements; 3. taking advantage of the development of biomaterials, microfabrication and tissue engineering to simultaneously evaluate and quantify multiple parameters instead of one at a time; 4. building patient-specific models.

### 3.1. From 2D Models to 3D Models

#### 3.1.1. Monolayer Culture and Scratch Assays

The simplest 2D model to study cell motility, especially with respect to cell-ECM interactions, consists of cells, cultured in monolayer, on glass or plastic slides [102]. ECM components can be used either as a coating or solubilized in the medium and the assay involves the observation of motility [102]. The random motility of cells can be analysed either by time lapse microscopy or the time required by confluent cells to close a “wound” scraped into the dish (scratch assay). Much of the evidence mentioned above, on the role played by ECM components, has been obtained using this approach, including the role of fibronectin, vitronectin, HA and MMPs [103,104,105]. Despite the simplicity of this model, its limitations are numerous [102]: 1. it considers motility and not invasion [8]; 2. it dismisses the role of stiffness, being glass and plastic slides characterized by an elastic module enormously higher than that of brain [106,107]; 3. It doesn’t consider the role of chemotactic gradients, hypoxia and interstitial fluid pressure [108,109]; 4. It is well known that cells in monolayer behave differently than cells in 3D [110,111].

#### 3.1.2. Transwell Assays

The transwell assay is another 2D model that can be used to study cell invasion [112]. This assay is based on the original Boyden assay system, consisting of two chambers separated by an insert with a porous membrane. Tumour cells are seeded in the upper chamber, while, in the lower one, serum or specific chemotactic factors are added to create a chemotactic gradient [113,114]. The assay, that evaluates the capacity of tumour cells to cross the insert, is versatile, since several parameters can be changed: the chemotactic gradient used to stimulate migration, the coating of the inserts and the pore size.

Regarding the chemotactic gradient, it has been demonstrated the motogenic action, often dose-dependent, of several factors, including PDGF, EGF and HGF as well as TGFα and FGF1 [115,116,117,118].

As insert coating, several protein mixtures have been used to mimic the matrix present in the tumour during dissemination [40], including basement membrane extract, hyaluronan, laminin and collagen I [58]. Usually, these matrices can act as chemoattractant via integrin signalling or through the release of growth factors embedded in the matrix itself [113]. One of the most used coatings is Matrigel^®^, containing a mixture of basement membrane extracts such as laminin, collagen IV and entactin, as well as several bound growth factors. Unfortunately, this protein mixture does not accurately reflects the GBM microenvironment and the variability among batches can give rise to divergent results [58]. Transwell assays are the most used to study the effects of therapeutic treatments. For example, it has been used to demonstrate that ionizing irradiation of PTEN null gliomas, U-251 MG and U-373 MG, determines an increased Matrigel™ invasion in association with an enhanced MMP-2 secretion [119].

Regarding pore size, it has been shown by Beadle’s group that the invasion mechanism is strictly dependent on the dimension of the barriers that cells must cross [14]. As mentioned before, the hydrodynamic model of cell invasion, depending on non-muscle myosin II, is activated only when cells cross 3 μm pore size and is absent when cells cross 7–8 μm pore size [14].

Transwell assays are widely employed because of the simplicity, the sensitivity and the possibility to take into consideration different parameters. However, they are an over simplification of the in vivo situation. Specifically, the 2D cell cultures can hardly reflect the presentation, organization and polarity of ECM proteins, hence they are not naturally suited to study the effects of matrix remodelling or cell-cell interactions, crucial events in tumour invasion [102]. For this reason, great efforts have been made to develop models able to investigate glioma cell-ECM interactions in 3D matrices in order to exploit the role played by matrix composition, stiffness and architecture in glioma progression.

### 3.2. 3D Models: Taking into Account Cell-ECM and Cell-Cell Interactions

#### 3.2.1. Ex Vivo Tumour Sections

Advances in in vivo intravital microscopy and electrophysiological study have allowed the use of ex vivo tumour sections to study invasion of tumour cells into brain slices. This model has been used to evaluate both perivascular [55] and parenchymal invasion [14,57]. Specifically, as above mentioned, Beadle and collaborators studied glioma cell migration in living brain tissues obtained from PDGF-driven rat gliomas. They demonstrated that the movement in the restricted interstitial spaces of the brain is similar to that of neural stem cells and consists in a first extension of a leading cytoplasmic process followed by an advancing movement of the cell body that is mediated by myosin II [55].

Regarding the perivascular invasion of glioma cells, Watkins et al. used brain slices of murine tumours obtained by orthotopic xenotransplantation of human cells. Through combined immunofluorescence and electrophysiological assays, they showed that the tone of vessels, in which astrocyte endfeets are displaced by perivascular cells, are not anymore controlled by astrocytes, causing a disruption of the astrocyte-mediated vascular coupling (gliovascular coupling) [55]. Conversely, the control of the vessel tone is assumed by perivascular glioma cells that acquire the ability to regulate either tumour invasion (by increasing the perivascular space by vasoconstriction), or growth (by favouring the tumour perfusion through vessel dilation) [55]. Interestingly, the same authors showed that, in vivo, the loss of contact between endfeet and blood vessels cause the opening of the blood-brain barrier (BBB) and that single glioma cells, even far away from the tumour mass, were capable of locally breaching the BBB and disrupting the gliovascular coupling [55].

#### 3.2.2. 3D Invasion Model

The initial approach, to create an environment in which not only one side of cells is in contact with the surrounding environment, is represented by seeding cells on top of a gel composed of collagen and adding a second layer above them, then counting migrated cells in both layers [120]. This experiment is also useful to better understand different associations between specific ECM components and cellular invasion; in fact, we can use and compare different upper and lower layers [120]. A subsequent evolution of this 3D invasion model is a 3D embedded invasion assay, where cells are seeded on a 96-well plate coated with a Matrigel or a collagen I/Matrigel mix (2:1) layer and in the middle of the well is created a cell-free gap with silicon stoppers, which is removed after 12 h. This approach is useful to monitor the invasion during the experiment and to observe changes in cellular morphology, therefore allowing not only a quantitative but also a qualitative assessment [121].

#### 3.2.3. Modified Transwell Assays

A variation of the original transwell assay is the so-called “trans-endothelial migration assay” [122], in which a confluent layer of endothelial cells are plated on the top of the membrane. Endothelial cells produce their ECM and establish cell-cell and cell-ECM junctions. It is useful to investigate intravasation [123] and extravasation [124] of tumour cells across the vasculature. It is known that glioma cells do not usually intravasate. However, glioma researchers exploited this model to test the chemo-attractive potentials of chemokines secreted by endothelial cells of the brain tumour vasculature (i.e., bradykinin) [13,125]. They used vitronectin and Matrigel, as coating of the transwell porous filters, to verify migration and invasion in response to various doses of the cytokine. They also showed that invasion is mediated by activation of MMP [13,125].

Another interestingly and more physiological alternative is represented by the “brain slice invasion assay” [126,127,128]. Slices of brain cortex are transferred into the upper chamber of a transwell insert and tumour cells (usually fluorescently labelled) seeded on top of the slices. This test consented a time-dependent quantification of glioma invasion into mammalian brain in vitro and the demonstration of a preferential invasion of white structures by glioma cells [126]. Additionally, it allowed to evaluate the effects of chemotactic factors and ECM components [128], as well as of drugs on the invasion process [126].

In these in vitro models, cells are almost always seeded as single cells. This limits the intercellular communication and therefore these assays don’t evaluate the process of detachment of tumour cells from the tumour mass. Additionally, these models are not able to take into account hypoxic and nutrient gradients present within the tumour. For these reasons, spheroids have been exploited.

#### 3.2.4. Spheroids

Spheroids are aggregates of cells grown in suspension or included in 3D matrices [10]. Therefore, they take advantage of both the cell propensity for self-aggregation and the ability of tumour cells to form spheroids in the absence of an adhesive substrate within a semisolid agar [129].

Weiswald et al. identified four spheroid models according to the generation and culture conditions [130]: 1. The multicellular tumour spheroid model (MCTS), obtained by culturing tumour cell lines in non-adherent conditions [131]; 2. Tumour spheres, obtained by culturing GSC in suspension in a serum-free medium [25]; 3. Tissue-derived tumour spheres (TDTS), composed by tumour cells after a partial tissue dissociation [132]; and 4. Organotypic multicellular spheroids (OMS) obtained by culturing ex vivo fragments of tumours without dissociation [133].

The most used spheroid models to study glioma invasion are MCTS [134,135,136,137] and OMS [133,138,139,140]. The choice of the model is important because different models are linked to different invasive capabilities [141].

OMS retain the same morphology of the tumour of origin, including the stromal component (e.g., macrophages and vessels associated with fibroblasts) [133]. Their genomic profiles are genetically stable and more similar to that of the tumour of origin than that of short-term primary cultures [142]. Importantly, OMS mimic the properties of their parent tumours. For example, it has been shown that OMS derived from low-grade glioma, with respect to those derived from high-grade gliomas, displayed, when co-cultured with foetal rat brain aggregates, less invasiveness [143]. Additionally, OMS obtained from different regions of the same glioblastoma are characterized by different migration and invasion capability, possibly reflecting the intratumoral heterogeneity [138].

MCTS, although time consuming with respect to 2D culture, recapitulate cell–cell and cell–matrix interactions between tumour cells and the microenvironment; additionally, larger spheroids are characterized by the presence of a necrotic core and both oxygen and nutrient gradients [10]. Accordingly, MCTS protein and gene expression profiles are more similar to those of the tumour of origin than that of the corresponding 2D cultures [10].

MCTS have been employed to study, upon treatment with specific chemotactic factors and therapeutic agents, the directional migration of tumour cells from the spheroid mass [134,136]. Specifically, by growing spheroids on plastic substrates and measuring the cell spread from the colonies formed upon adhesion, it has been assessed the strong effect of EGF [135,140] and bFGF [140] on cell invasion. Using the same assay, Wild-Bode’s group showed that sub-lethal irradiation of glioma cells induces an increase in the migration distances due to an enhanced expression and activation of MMP-2, MMP-9 and MT1-MMP [137].

More recently, a fifth model has been developed—i.e., a 3D organoid culture, obtained by culturing tumour cells in matrigel for months—starting either from a glioma culture or from dissociated brain tumours—and allowing the development of complex structures mimicking tumour development [141]. Organoids are characterized in vitro by the presence of both hypoxic gradient and cell heterogeneity [141]. Although not yet tested in vitro in invasion assays, once injected in vivo they gave rise to tumours strictly resembling the in vivo invasion pattern of the tumour of origin [141].

### 3.3. Engineered Models

TDTS and OMS are relevant models for mimicking tumours but besides being time consuming, they are not susceptible to transfection and the standardization of the models is difficult. It is particularly challenging the control of the single parameters that can play a role in tumour invasion, such as stiffness, ECM composition, cell-ECM interaction, oxygen and nutrient gradients and migration through confining tracks [11,32,113,144].

Advances in microfabrication, new biomaterials, surface science and tissue engineering have allowed the development of in vitro model able to overcome some of these issues [11,102]. Specifically, engineered models rely on the ability to manipulate the architecture and the molecular composition of scaffolds in order to create a microenvironment in which it is possible to evaluate glioma cell response to extremely specific and controlled stimuli.

In this regard, several strategies have been exploited to create tumour in vitro models, such as biomimetic hydrogels, microchannel devices, grooved substrates, microcontact-printed and micropatterned lines, vertical confinement devices and micropost arrays [144]. So far glioma invasion research has relied mainly on the use of biomimetic hydrogel, i.e., gels constituted by extracellular matrix proteins or chemically produced polymers [144].

Here, we will review the methods used to assess the different aspects of glioma invasion in 2D and 3D models.

#### 3.3.1. Stiffness, Confinement, ECM and Chemotactic Gradients in 2D

The simplest method used to evaluate the role of stiffness and ECM composition relies on engineering substrata with controlled elastic modulus and specific biochemical functionalization [102]. This is obtained by using polymeric hydrogel matrices, usually based on cross-linked polyacrylamide (PAA) [51,145,146,147], functionalized by adsorption or covalent binding of the ligands of interest [148,149]. Using this approach, Kumar’s group demonstrated that an increase in ECM stiffness induces both proliferation and motility of glioma cells [51] and that the signalling pathways involved in the mechanotransduction include non-muscle myosin II [51], α-actinin [150], talin [151] and Rho GTPase RhoA [152] and are often driven by EGFR, CD44 and integrins [37,153,154].

An increase motility could also be obtained by taking advantage of physical topography and confinement of cells [155]. Control of topography and confinement have been made possible by the introduction of microfluidics systems [113]. These latter are mainly constructed by polydimethylsiloxane (PDMS) via soft lithography [156] and their transparency and thinness allows the analysis by time lapse-microscopy [113]. Using PDMS, it was possible to obtain devices that can mimic the migratory path present in the brain (e.g., white matter tracts) through the realization of channels of small diameter. Moreover, microchannels could be coated and/or filled with ECM components, either in 2D or 3D, to evaluate the effect of the different matrices on motility and measuring the traction forces exerted by cells during migration [157,158,159]. Specifically, cells cultured in micron-sized channels [146] or on substrates with aligned nanofibers [160,161] showed an increased motility. In the first case, it was also possible to demonstrate that the effect of the confinement was independent from that of stiffness [146].

Regarding functionalization of the substrata [162,163,164], it has been investigated the role of integrins and CD44 on tumour cell motility [147,163,165], showing how HA can increase glioma cell motility [165]. Moreover, more recently, researchers focused on the development of protocols to generate cytokine gradients mimicking the ones present in vivo in the brain tumours. Several systems have been developed, using flow-based gradient generators, diffusion-based gradient generators, hybrid generators, as well as passive diffusion [166,167,168].

#### 3.3.2. Engineered 3D Models

The advantage of engineered 3D culture resides in the possibility to observe a more physiological behaviour of cells that can move degrading and remodelling the scaffold in any direction, even when the scaffold mimics a confinement situation [11,102,111,166,169,170]. Moreover, 3D models can incorporate controlled oxygen and nutrient gradients and can be seeded with different cell types. Additionally, with respect to “natural” 3D models, they are engineered with the objective to follow in real time, by time lapse microscopy, the invasion process [11,102,111,166,169,170].

##### Stiffness

Stiffness in 3D hydrogel models is usually obtained by modifying either the crosslinking density or the polymer concentration [11]. Interestingly, GBM cells included in 3D matrices showed a different behaviour with respect to those cultured in 2D scaffolds [51,165,171]. Specifically, cell motility resulted to be inversely related to the stiffness [51,165,171]. Contrasting results were instead obtained concerning MMP secretion. For example, HA-matrices resulted to either enhance or decrease MMP9 secretion [153,154,172]. The reason has been attributed to the fact that, when using bioactive molecules as a scaffold (e.g., HA), it is difficult to discriminate the singular contribution of stiffness and biochemical stimuli on cell invasion [11].

##### ECM Composition

In order to mimic in vitro the 3D microenvironment present in gliomas, different ECM-components have been utilized to construct 3D hydrogels, such as HA [153,154,162,173], chondroitin sulphate [174], chitosan [175,176] and collagen/gelatine [153,154,174,177,178,179], being the HA-ones the most used. In these 3D hydrogels, increasing concentrations of HA or chondroitin sulphate were associated with increased migration of commercially available and patient-derived GBM cell lines [165,180] and increased production of matrix degrading enzymes such as hyaluronidases [177], MMP [153,154] and HIF [153,154]. These 3D models, applied to patient derived cells, were also more predictive of therapy response [181].

Since HA is per se not able to bind cells, gelatine [153,154] or collagen I are added to offer integrin-binding sites [174,177]. However, in hybrid HA/collagen I scaffolds the described effects on cell migration were conflicting [174,177], remarking the possible crucial effect of each single parameter, including, for example, the cell culture methods (single cells vs. spheres) [11]. As an alternative, RGD peptides [165] or k-elastin [182] were included in the 3D HA-scaffold, increasing glioma cell adhesion and MMP production [182].

Additionally, as previously mentioned, 3D scaffold can be added with cytokines and growth factors. In this way, the pro-migratory effect of EGF [183] and the role played by heparin-cytokines interaction [184] have been shown.

#### 3.3.3. Migration along Constrained Paths

##### Parenchyma Invasion

As mentioned, the migration of glioma cells through tight spaces requires both the production of enzymes able to degrade the ECM and the ability to squeeze through pores whose diameter can be less than that of cell nuclei (see Section 2.1).

3D scaffold can mimic these aspects. However, in most cases, because of the fabrication modalities, it is impossible to separate the effects of stiffness, ECM concentration and sponginess [11]. This can explain the contrasting results often obtained [11]. In fact, stiffness is frequently controlled by modulating polymer concentrations and this is paralleled by changes in pore diameters. Moreover, if the scaffold is obtained using HA, increasing the stiffness means increasing the concentration of bioactive molecules [11]. Therefore, bioengineers are now working to obtain systems in which all these aspects are decoupled and quantifiable.

For example, 3D PEG-scaffolds were fabricated with incorporated sites sensitive to MMP degradation, showing an increased invasion ability of glioma cells [176,179]. HA-scaffolds are instead, per se, able to measure the ability of cell lines to produce hyaluronidases.

Only a few scaffolds able to decouple stiffness, porosity and biochemical stimuli have been tested for glioma cell invasion. For example, a 3D scaffold was fabricated keeping constant the concentration of collagen I and adding increased agarose concentrations, to increase the stiffness [185]. In this platform, the increased scaffold stiffness, was paralleled by a reduction in the migration speed of glioblastoma cell lines [185].

3D scaffolds have been also fabricated to reproduce chemotactic as well as stiffness gradients, showing how both can direct migration [154,163].

Although not yet used for glioma, scaffold with oxygen gradients have been developed and can, in the future, give information also on this aspect [186].

##### Perivascular Invasion

Glioma cells do not intravasate into the blood vessels, such as most tumour cells but they are attracted on the external wall of the vessels and migrate along these ones [13]. Designing in vitro assays to reproduce and analyse this process, requires a greater effort due to its peculiarity [187]. To this aim, researchers can adopt organ-on-a-chip platforms, organogenesis-based models or hybrid models [188,189]. The first models rely on microfabrication and microfluidics technologies to reproduce 3D vessels, structurally and functionally similar to the real ones, to better study interactions between tumour cells and vasculature in a realistic dimension [187]. The second ones involve the self-organization of primitive cells into structures recapitulating vessels [187]. Hybrid models consist in culturing cells on organ-on-a-chip devices [187].

The simplest version of these platforms is constituted by microvessels built by using PDMS cylindrical templates embedded in an ECM. After removal of the channel scaffolds, endothelial cells are seeded in their internal surface and lined along channels, originating a 3D model of microvessels. Tumour cells and any other type of cells residing in the tumour microenvironment can be introduced in the ECM around the vessels to study tumour-endothelial cell interactions [190,191]. PDMS microdevices could mimic in vivo migratory routes of cancer cells along channels and cell motility can be monitored by time-lapse imaging. Moreover, it is possible to change the ECM composition, reproducing as much as possible the in vivo environment [192].

As an evolution of the above described model, 3D microfluidic models have been realized. An exhaustive description can be found in [169,188]. Briefly, the microfluidic models consist of two different channels (frequently in PDMS, embedded with gelatine, as templates), where it is possible to seed, separately, tumour and endothelial cells. Channels are interconnected by a 3D ECM, in which tumour cells invade in response to externally applied gradients of stimuli. This model represents a platform that can be studied by high resolution live cell imaging [193].

Despite the benefits deriving from the use of the above described devices, they show one disadvantage, that is the generation of vessel with limited diameter ranges (50 μm) because endothelial cells are seeded in predefined cylindrical template channels. This condition creates a vasculature with linear geometry, not similar to the random and complex networks of smaller vessels/capillary that characterize tumours.

Miller and co-workers described, instead, the creation of a bio-compatible scaffold characterized by cylindrical network of filaments, lined with endothelial cells, encapsulated inside an ECM along the living cells of interest [194]. As template of channels, filaments in lattice are assembled inside a scaffold composed of a carbohydrate glass, derived from solving in water a mixture of sucrose and glucose. This device: allows the control of diameter and geometry of the vascular network; is compatible with different cell types and ECM; can be perfused and represents a framework easy to monitor [194].

Although promising, these devices have not been used to study glioma invasion yet.

#### 3.3.4. Interstitial Flow

Qazi et al. reported a novel microfluidic device to study the influence of fluid shear stress on the migratory activity of glioma cells [195]. This 3D Modified Boyden chamber model was designed to mimic the fluid dynamic microenvironment. The use of this model showed that the motility of some but not all, commercially available glioma cell lines were reduced by fluid shear stress. This latter acted via mechanotransduction by modulating MMP activation and expression [195].

Conversely, as previously mentioned, Munson’s group engineered a 3D tissue model showing that flow can increase invasiveness of patient-derived glioma cells lines. This effect resulted to be mediated either by CD44-mediated mechanotransduction or autologous chemotaxis via CXCR4–CXCL12 signalling. Importantly, patient-derived cells were heterogeneous in terms of populations acting through these two different pathways. Additionally, radiotherapy resulted to increase the invasiveness of glioma cells responsive to CXCR4–CXCL12 signalling [59].

These contrasting results obtained using different models, once again underline the difficulty of modelling in vitro all the different parameters that come into play in vivo.

#### 3.3.5. Cell-Cell Interaction

The purpose of these assays is to evaluate the interaction of cells within the glioma microenvironment (e.g., tumour cells, astrocytes, microglia), keeping into consideration both paracrine factors and cell-to-cell contacts in 3D. For example, it has been shown that co-culturing GBM cell lines with immortalized astrocytes, within spheroids, protects tumour cells from temozolomide-induced apoptosis [196]. 3D co-cultures between fluorescently labelled GBM cell lines and immortalized microglial cells have also been optimized utilizing a Matrigel scaffold and showed how microglial cells are able to greatly increase glioma cell invasiveness [197].

Although promising, these co-culture assays took advantage of the use of commercially available cell lines, while no information regarding the use of patient-derived cell lines are available. The major problem in co-culturing different cell types, including patient-derived cells, resides in the distinctive culture conditions that different cell types require. The development of culture conditions able to support the co-culture of different cell types represents an important challenge for the future. However, one possible way to partly overcome this problem is using synthetic peptides, such as RGD or N-Cadherin, within 3D systems mimicking cell-to-cell contacts [198]. Although these devices can give insights into the single contribution of specific signalling pathways, they are unable to mimic the dynamic relationships acting in vivo.

### 3.4. Towards Patient-Based Assays

The choice of the cells to be used in the in vitro assays represents a key point. In this regard, continuous, commercially available, human tumour cell lines (e.g., A172, LN229, SF268, U87MG, U118MG and U138MG) have been widely used [199,200]. Although these cell lines are easy to expand, both in vitro and in vivo pre-clinical screening models, they often failed in predicting the outcome of clinical trials [200]. Frequently, the genetic aberrations found in the continuous human cell lines differ from the ones characterizing the human tumours [199]. Moreover, the xenografts obtained by injecting them in vivo do not phenotypically resemble human tumours [199]. These observations suggested carefulness in transferring the relevance of what observed in cell lines to human primary tumours [199] and underlined the critical need for new and more biologically and clinical relevant in vitro models [201]. With this purpose, short-term cultures have been obtained culturing freshly isolated cells as monolayers in serum-enriched medium [202,203]. However, whether these cell lines can be considered truly representative of the original tumour has been questioned [199]. A significant breakthrough has been represented by the possibility to isolate and in vitro expand from gliomas, the so-called glioma stem cells (GSC) or glioma-initiating cells [25,26]. These latter represent a rare fraction of tumour cells endowed with stem cell properties and therefore able to self-renew and, once injected into immunocompromised mice, to originate a tumour that exactly recapitulate the tumour of origin. GSC represented a ground-breaking scientific discovery because: 1. It identified, within gliomas, the same hierarchy present in normal tissues [204]; 2. It established a new target in cancer treatments [25,26,205]: therapies unable to kill this rare population, often intrinsically resistant to many drugs, are destined to fail [206]; 3. Finally, xenografts originated by injecting GSC shared the same genetic landscape and phenotype of the original tumours, being a bona fide phenocopy of the patient’s tumour [25,26]. This would allow overcoming some of the crucial limitations presented by human continuous cell lines. Indeed, the possibility to obtain, for each different patient, an in vitro model genetically and phenotypically representative of his/her tumour, makes possible to move toward a concept of personalized therapy for individual tumours. This requires isolating stem cells from each tumour patient, to expand them at an adequate number within a clinically acceptable time. Unfortunately, the two classical methods of isolation of GSC do not completely fulfil these criteria [25,26]. The fraction of cells expressing CD133, the most common GSC marker, is usually low; conversely, neurosphere formation is a time consuming procedure, succeeding only in a fraction of high-grade glioma samples [207]. Modifications of the original protocols [208] and the optimization, by Pollard’s group, of a novel procedure based on culturing GSC in adhesion on laminin-coated dishes [205], can overcome some of these restrictions.

However, as postulated by Hanahan and Weinberg, cancer is not simply constituted by proliferating cancer cells but it consists of different cell types involved in heterotypic interactions [27,29]. Importantly, this tumour-associated stroma is not an inert witness but it plays an active role in tumorigenesis contributing to the development of the hallmark properties of cancers [27], such as sustaining tumour proliferation, inducing angiogenesis, avoiding immune destruction, deregulating cellular energetics, inducing invasion and metastasis [209,210,211]. Additionally, tumour stroma is now considered endowed with a prognostic and a predictive function and it represents a novel targeting opportunities [212,213,214]. Therefore, the possibility to create an in vitro model representative of the tumour stroma can get new insight into tumour biology and represents another key element for a patient-based approach.

Regarding gliomas, xenotransplantation experiments employing human glioma cell lines have suggested that astrocytes in the vicinity of glioma cells can be activated and facilitate tumour invasiveness [215]. In addition, genetic fate mapping studies have shown that reactive glia could acquire a stem cell potential [216,217]. Similarly, PDGF-induced gliomas arising in both adult and neonatal rats contain normal stem and progenitor cells “recruited” into glioma mass and induced to proliferate, supporting the hypothesis that proliferative stem-like portions of the tumour can arise from normal progenitors [218].

In this regard, we easily isolated from human low- and high-grade glioma a population of stem cells, named glioma associated stem-cells (GASC) characterized by an undifferentiated mesenchymal phenotype, clonogenicity and multipotency, being able to differentiate into multiple ectodermic (glial, oligodendroglial and neuronal-like cells), mesodermic (endothelial-, osteoblast- and myocyte-like cells) and endodermic (e.g., hepatocyte-like cells) derivatives, although with a variable efficiency [101,219,220]. Although devoid of in vivo tumour-initiating properties and of the genetic aberrations characterizing the tumour of origin, GASC were characterized by the ability to grow in an anchorage independent way and to support the biological aggressiveness of tumour cells, including their motility, through the release of exosomes [101]. Similar results were obtained by the Lang’s group, describing a population of glioma-associated mesenchymal stem cells [100,221]. Importantly, it has been demonstrated that GASC were endowed with a prognostic potential in low-grade glioma, being a score based on their phenotype, the strongest independent prognostic factors of overall survival and malignant progression free survival [101].

Since GASC-derived exosomes were able to modify the motility of both immortalized glioblastoma cell lines and patient-derived GSC, by using time-lapse microscopy as well as atomic force microscopy and single cell force spectroscopy, it was demonstrated that, independently from the grade of the glioma of origin, GSC are softer than GASC, in agreement with their neoplastic features [222]. Additionally, using patient derived cells, it has been demonstrated that the adhesion strength of GSC on GASC appears to be dictated by the grade of the tumour of origin, being the strength significantly higher for GSC/GASC pairs derived from low-grade gliomas, with respect to the pairs derived from high-grade gliomas [222]. This is in line with the fact that high-grade glioma cells are characterized by a more infiltrative nature with respect to the low-grade glioma ones. What is even more interesting is the fact that when GSC from high-grade glioma were cultured, in parallel, on GASC derived from low-grade and high-grade gliomas, they tightly adhere on GASC from low-grade gliomas but not on those from high-grade gliomas. This suggests that the grade of GASC (that is the tumour microenvironment) plays a key function in modulating cancer cell adhesion, thus possibly affecting glioma cell migration, invasion and thus cancer aggressiveness [222]. More recently it has also been shown the critical role played by NF-kB in the tumour supporting function of GASC [223].

Although these evidences underline the enormous clinical potential of using patient-derived cells and possibly tumour cells together with patient-derived TME cells, their use in 3D in vitro assays is still hampered by the difficulties in co-culturing cells requiring different media. Additionally, the TME is constituted not only by stem cells, surely the more aggressive and powerful tumour sub-population but also by differentiated cell types. And again, these sub-types can be obtained starting from undifferentiated cells but this process requires specific differentiation inducing conditions, not easy to simulate in complex 3D in vitro assays.

## 4. Summary and Future Perspectives

The final aim of studying glioma invasion is to better understand the mechanisms underlying it in order to develop novel therapeutic strategies. Table 2 summarizes some of the therapeutic approaches that have been tested in vitro to interfere with glioma invasion. These latter include strategies to target the cytoskeleton, cell adhesions, matrix degradation enzymes, as well as inhibitors of receptor tyrosine kinases and chemokine receptors, ion and water channels, transcription factors such as HiF-1α and pro-tumorigenic inflammation [224]. However, despite the high number of drugs tested, no anti-invasion molecules have been yet approved for clinical use. For example, clinical trials using MMP inhibitors such as marimastat and prinomastat were terminated for unwanted side-effects [225], while the integrin inhibitor cilengitide failed to show a clinical benefit when added to standard therapy [226]. Clinical trials using PI3K inhibitors, although still ongoing, are showing limited effects on tumour regression at tolerated doses [227]. Similarly, clinical trials using small molecules kinase inhibitors still did not change the clinical practice [228]. Encouraging, although variable, results are deriving from targeting hypoxic cancer cells and/or HIF [229]. Many are the reasons of the failure. To be effective, drugs must cross the blood-brain-barrier, specifically target tumour cells and be devoid of too severe off-targets effects. However, if we carefully look to the in vitro test used to predict the ability of the drugs to hamper glioma invasion, we can observe that most of them were scratch and transwell assays, perhaps the less capable to mimic the in vivo situation.

Advances in biological knowledge paralleled by the progresses obtained in the field of biomaterial and tissue engineering have amplified the offer of in vitro tests that can more and more resemble the in vivo state.

The results obtained so far have certainly helped to shed light on this phenomenon but have also highlighted how different in vitro models can lead to different results. This happens not only when switching from 2D models to 3D models but also within the same category of assays. This, once again, shifts the focus on the importance of the research of the factors that play a key role in vivo and on the ability to control them in the in vitro assays. But it is not enough, since it is then essential to confirm, in vivo, what emerges from the in vitro tests.

In this regard, a possible help could derive from the application of mathematical simulation to create predictive models starting from the multiple information obtained possibly overcoming the difficulties associated with in vivo research and the considerable limitations of in vitro research [230].

Finally, co-cultures and the use of patient-derived cells can contribute to make these in vitro models even more clinically relevant. As mentioned, this will require the development in the future of in vitro systems capable of satisfying the specific culture needs of different cell types.

In conclusion, the in vitro models helped to broaden our knowledge on the phenomenon of glioma invasion. However, it will be necessary to learn a lot more because these in vitro assays can become clinically relevant predictive models.

## 5. Methods

To identify the scientific literature regarding glioma invasion and in vitro models we reviewed the literature in the PubMed database. We focused on publications written in English and published until December 2017. As search terms, we used “invasion mechanisms”, “glioma invasion”, “in vitro model of invasion”, “glioma model of invasion”, “spheroids”, “organoids”, “GSC and invasion”, “engineering and invasion”, “in vitro model of tumour microenvironment”, “engineering tumour microenvironment”, “microenvironment and invasion”, “glioma and invasion and drugs”. Due to the large number of papers, we were not able to cite all individual references. We apologize to all authors whose important publications are not cited.

## Figures and Tables

**Table 1:** Major results obtained by studying specific aspects of glioma invasion in 2D and 3D models.

Model	Mechanism Studied	Significance	Reference
**2-D Models**			
Monolayer culture on glass or plastic slides (scratch assays)	Cell motility	Used to define the effect of several ECM components and soluble factors on glioma cell motility.	[35,36,37,38,39,40,41,42,43,44,45,46,47,48,103,104,105,113]
Transwell migration	Cell motility and cell invasion depending on:		
	Chemotactic gradient	Used to define factors able to favour or inhibit glioma invasion.	[115,116,117,118]
	Insert coating	Used to assess the role of ECM components on cell invasion.	[40,58,113]
	Pore size (e.g., 8 μm vs. 3 μm)	Glioma cells requires myosin II only when migrating through 3 μm in diameter pores.	[14]
**3D Models**			
Modified transwell migration	Trans-endothelial migration assay: effect of endothelial cells on glioma invasion	Role of bradykinin in the perivascular invasion.	[13,125]
	Brain slice invasion assay: invasion of brain tissue slices	Effects of ECM components, soluble factors and drugs on glioma cell motility.	[126,127,128]
Spheroids	Multicellular tumour spheroids (MTS)	Effects of motogenic substances and irradiation on migration upon adhesion on plastic substrates.	[134,135,136,137]
	Organotypic multicellular spheroids (OMS)	Role of different ECM components on cell migration from patient derived spheroids.	[133,138,139]
Ex vivo tumour sections	Tumour slices of PDGF-driven rat gliomas: glioma migration in living brain tissue through extracellular spaces that are in the submicrometer range	When invading the extracellular spaces, glioma cells squeeze through pores smaller than their nuclear diameter and this process requires myosin II.	[14]
	Tumour slices of brains xenotransplanted with human tumour cells: perivascular invasion of glioma cells	Perivascular glioma cells disrupt both astrocyte–vascular coupling and the blood–brain barrier.	[55]
**Engineered Models**			
*2D*	Stiffness: substrata with controlled elastic modulus	An increase in ECM stiffness induces motility of glioma cells. Mechanotransduction involves non-muscle myosin II, α-actinin, talin and Rho GTPase RhoA.	[51,150,151,152]
	Physical topography and confinement of cells	Cells cultured in micron-sized channels or on substrates with aligned nanofibers showed an increased motility.	[146,157,158,159,160,161]
	ECM composition and chemotactic gradients	Definition of the role of ECM components (e.g., integrins and CD44/HA) and chemotactic gradient on tumour cell motility.	[147,162,163,164,165,166,167,168]
3D	Stiffness	Cell motility resulted to be inversely related to the stiffness. However, regarding MMP secretion, HA-matrices resulted to either enhance or decrease MMP9 secretion.	[153,154,165,171,172]
	ECM composition	Role of different ECM-components, utilized to construct 3D hydrogels, such as HA, chondroitin sulphate, chitosan and collagen/gelatine, on migration of commercially available and patient-derived GBM cell lines. Evaluation of the underlined pathways (e.g., production of matrix degrading enzymes such as hyaluronidases, MMPs and HIF). Evaluation of the pro-migratory effect of EGF and the role played by heparin-cytokines interaction.	[153,154,162,165,173,174,175,176,177,178,179,180,181,183,184]
	Migration along constrained paths	Mechanisms underlying the parenchyma invasion.	[154,163,176,179,185]
		Perivascular invasion: 3D models available but not yet tested with glioma cells.	
	Interstitial flow	Contrasting results showing the pro-migratory and anti-migratory effects of the interstitial flow. Role of CD44-mediated mechanotransduction and autologous chemotaxis via CXCR4–CXCL12 signalling.	[59,195]
	3D cell-cell interaction	Anti-apoptotic effect of astrocytes co-cultured with tumour cells.	[196]
		Pro-migratory effects of microglial cells co-cultured with tumour cells.	[197]

**Table 2:** Use of in vitro models to assess new therapeutic strategies interfering with glioma invasion.

Model	Drugs	Mechanism of Action	Reference
**2-D Models**			
Scratch assay/Transwell migration	Bumetanide	The inhibition of the Sodium-Potassium-Chloride Cotransporter Isoform-1 (NKCC1) affect cell motility only when cells had to undergo volume changes during migration.	[231]
	Fluvoxamine	Selective serotonin reuptake inhibitor (SSRI) disrupting actin polymerization and inhibiting glioma motility and invasion.	[232]
	Glycogen synthase kinase-3 β (GSK-3β) inhibitors	Glycogen synthase kinase (GSK) 3beta inhibitors are able to attenuate glioma invasion in vitro and in vivo.	[233]
	Blebbistatin and Rho-associated kinase (ROCK) inhibitor Y-27632	Inhibitors of non-muscle myosin (NMMII) IIA and IIB affect glioma cells migration through 3 μm in diameter pores (confined spaces).	[14]
	PIK3CA or PIK3R1 abrogation by lentiviral-mediated shRNA	Abrogation of PIK3CA or PIK3R1 reduces glioma invasion and motility in vitro.	[234]
	Sulfasalazine	The block of the system x_c_ inhibit glutamate release thus reducing chemotactic invasion and scrape motility assays.	[235]
	HIF-1α abrogation by lentiviral-mediated shRNA	Knock down of HIF-1α in glioma cells significantly impairs their migration in vitro and in vivo.	[64]
	Indomethacin	This non-steroidal anti-inflammatory drug is able to reduce glioma cell invasion mediated by MMP-2 and MMP-9.	[236]
	Cyclosporin A (CsA)	CsA impairs migration and invasion of human glioblastoma cells by downregulation of Akt phosphorylation.	[237]
	Cilengitide	Integrin (αvβ3 and αvβ5) inhibitor able to reduce glioma invasion in vitro.	[238]
	Cholorotoxin	The inhibitor of the chloride channel-3 (ClC-3) partly inhibits glioma migration by disrupting volume changes.	[239,240]
	Disulfiram	NF-kB inhibitor able to reduce glioma cell invasion.	[241]
	Imipramine Blue	It inhibits NADPH oxidase-mediated reactive oxygen species generation and modifies the expression of actin regulatory elements reducing glioma invasion in vitro and in vivo.	[242]
**3D Models**			
Modified transwell migration	Icatibant	B2 bradykinin receptor inhibitor acting on the perivascular invasion.	[13]
	Autocamtide-2 related inhibitory peptide (AIP)	CaMKII (Ca^2+^/calmodulin-dependent protein kinase II) inhibitor are involved in the hydrodynamic model of cell invasion.	[125]
	Vincristin and paclitaxel	Cytoskeleton destabilizers are able to inhibit glioma cell invasion in a dose dependent manner.	[126]
Spheroids	Antibodies to the EGF receptor	Effects of antibodies on proliferation and migration in multicellular tumour spheroids.	[135]
	Lithium chloride (LiCl) and Bio-Indirubin (BIO)	Glycogen synthase kinase-3 β (GSK-3β) inhibitors are able to inhibit cell invasion in multicellular tumour spheroids.	[243]
	Methotrexate (MTX) and trimetrexate (TMX)	Anti-folate agents affect glioma invasion in 2D culture but not in multicellular tumour spheroids.	[244]
	Downregulation of cathepsin B, uPA, uPAR and MMP-9 using small, interfering, hairpin RNA (siRNA)	Retardation of glioma cell invasion in vitro and in vivo.	[245,246]
Ex vivo tumour sections	Blebbistatin and Rho-associated kinase (ROCK) inhibitor Y-27632	Inhibitors of NMMIIA and IIB are involved in glioma cells migration in living brain tissue through confined spaces.	[14]
**Engineered Models**			
2D	Blebbistatin and Rho-associated kinase (ROCK) inhibitor Y-27632	Pharmacological inhibition of NMMII or its upstream regulator ROCK blunts the sensitivity of glioma cells to ECM stiffness and renders this relationship insensitive to matrix confinement.	[51,146]
	Suppression of α-actinin-1 and α-actinin-4 by small interfering RNA (siRNA)	Disruption of α-actinin-1 and α-actinin-4 reduces cell motility and the sensitivity of glioma cells to ECM stiffness	[150]
	Tyrphostin Triciribine Wortmannin	Stiffness-dependent glioma cell behaviour is altered by treatment with EGFR inhibitor (Tyrphostin), Akt inhibitor (Triciribine) and PI3 Kinase inhibitor (Wortmannin).	[147]
3D	GM6001 BB94 TIMP1 MMP12 function blocking antibody	The tenascin-C–mediated invasiveness can be blocked by broad-spectrum metalloproteinase inhibitors (GM6001 and BB94). However, this effect did not involve MMP-2 and MMP-9, as shown in 2D assays but MMP12.	[179]
	GM6001	The broad-spectrum MMP inhibitor is able to interfere with the EGF-induced glioma cell migration in 3D.	[183]
		The broad-spectrum MMP inhibitor is able to interfere with the flow-modulated motility.	[195]
	AMD 3100	Non-competitive CXCR4 inhibitor interfering with the interstitial flow enhanced invasion.	[58]
